# Regulatory Mechanism of MicroRNA Expression in Cancer

**DOI:** 10.3390/ijms21051723

**Published:** 2020-03-03

**Authors:** Zainab Ali Syeda, Siu Semar Saratu’ Langden, Choijamts Munkhzul, Mihye Lee, Su Jung Song

**Affiliations:** 1 Soonchunhyang Institute of Medi-bio Science, Soonchunhyang University, Cheonan 31151, Korea; zainabali.10125@gmail.com (Z.A.S.); langdensema@gmail.com (S.S.S.L.); choijamtsm@gmail.com (C.M.); 2 Department of Integrated Biomedical Science, Soonchunhyang University, Cheonan 31151, Korea

**Keywords:** microRNA, microRNA biogenesis, cancer, genetic alterations, epigenetic modification, post-transcriptional regulation

## Abstract

Altered gene expression is the primary molecular mechanism responsible for the pathological processes of human diseases, including cancer. MicroRNAs (miRNAs) are virtually involved at the post-transcriptional level and bind to 3′ UTR of their target messenger RNA (mRNA) to suppress expression. Dysfunction of miRNAs disturbs expression of oncogenic or tumor-suppressive target genes, which is implicated in cancer pathogenesis. As such, a large number of miRNAs have been found to be downregulated or upregulated in human cancers and to function as oncomiRs or oncosuppressor miRs. Notably, the molecular mechanism underlying the dysregulation of miRNA expression in cancer has been recently uncovered. The genetic deletion or amplification and epigenetic methylation of miRNA genomic loci and the transcription factor-mediated regulation of primary miRNA often alter the landscape of miRNA expression in cancer. Dysregulation of the multiple processing steps in mature miRNA biogenesis can also cause alterations in miRNA expression in cancer. Detailed knowledge of the regulatory mechanism of miRNAs in cancer is essential for understanding its physiological role and the implications of cancer-associated dysfunction and dysregulation. In this review, we elucidate how miRNA expression is deregulated in cancer, paying particular attention to the cancer-associated transcriptional and post-transcriptional factors that execute miRNA programs.

## 1. Introduction

Normal cells can be progressively developed to the neoplastic stage by acquiring multistep processes of tumorigenesis, and they become malignant, which, in turn, leads to initiate cancer. The study of molecular mechanisms of the initiation and progression of cancer has become a core of cancer research, which can provide a scientific basis for developing the prevention and treatment strategies of cancer patients. Alterations in gene expressions associated with cancer are caused by the dysfunctions of various types of regulators, among which, microRNAs have received great attention in the past decades. MicroRNAs (miRNAs) are ~22 nt small noncoding RNAs that are known to play an important role in the post-transcriptional regulation of messenger RNA (mRNA). miRNAs are typically generated from the nascent primary miRNA (pri-miRNA) transcripts through two sequential cleavage events. The pri-miRNA is initially processed by DROSHA in the nucleus, which releases a hairpin-shaped precursor (pre-miRNA). Pre-miRNA is exported from the nucleus to cytoplasm by exportin 5 (XPO5) and cleaved by DICER. The resulting small RNA duplex is loaded onto the Argonaute (AGO) protein, which preferentially retains only one strand of mature miRNA by removing the other strand [1]. The miRNA-loaded AGO associates with other cofactors, including GW182 (also known as TNRC6A), and constitutes the effector complex called the RNA-induced silencing complex (RISC) [2]. The miRISC (miRNA-induced silencing complex) induces the decay of mRNA and translational suppression through the interaction with the complementary sequences in the 3′-untranslated region (3′-UTR) of target gene mRNA [3,4,5]. The miRNAs target a majority of mRNAs, enabling them to have important regulatory roles in diverse physiological and developmental processes [6]. In particular, miRNA-mediated gene expression control is critical for the cellular response to the environmental stresses, such as starvation, hypoxia, oxidative stress, and DNA damage, thereby being implicated in human diseases such as cancer. Indeed, numerous miRNAs can function as oncogenes (referred to as “oncomiRs”) or tumor suppressors (“oncosuppressor miRs”), and dysregulation of miRNA expression is closely associated with cancer initiation, progression, and metastasis [7,8]. In this review, we summarize how miRNA expression is deregulated in cancer, paying particular attention to the cancer-associated transcriptional and post-transcriptional programs, including transcriptional control, epigenetic methylation of miRNA loci, and dysregulation of the mature miRNA biogenesis pathway. We will further discuss the major genetic and epigenetic mechanisms involved in upregulating or downregulating miRNA expression, in an attempt to elucidate which elements are key to this process in cancer pathogenesis.

## 2. miRNA Deregulation in Cancer

In the past decades, miRNAs have been demonstrated to be extensively deregulated in human cancers, highlighting their important role in tumor onset, growth, and metastasis. Lu et al. demonstrated the profiling of 217 mammalian miRNAs from normal and human cancer samples and found that miRNA expression is globally suppressed in tumor cells compared to normal cells [9]. In addition to the global downregulation of miRNA expression, Volinia et al. presented the differentially expressed miRNAs in 540 solid tumor samples, indicating that specific alterations of individual miRNA expression were also apparent in tumors [10], since miRNA expression has been deregulated during cancer progression, creating an explicit expression pattern; for instance, the level of miR-21 expression is higher in early stage of diffuse large B-cell lymphoma (DLBCL) than in later stages [11]. Interestingly, some miRNAs are packed into a vesicle-like structure called exosomes for secretion, which can circulate throughout the body and can act differentially in a tissue-dependent manner. These include miR-21, the miR-200 family, and the miR-17∼92 cluster, and these exosomal miRNAs have been proved to be functionally implicated and clinically relevant in cancer [12]. The tumor microenvironment (TME) modulation accounts for the patient heterogeneity of treatment responses [13]. Therefore, miRNA-based exosomes represent one of the dynamic facets of the tumor microenvironment, and exosomal miRNAs in the TME may profoundly impact on tumor progression and therapeutic efficacy. 

The role of specific miRNAs in cancer was firstly appreciated by Calin et al. [14]—the deletion of miR-15 and miR-16 genomic loci in the majority of samples from chronic lymphocytic leukemia patients. Cimmino et al. further confirmed that miR-15 and miR-16 induce apoptosis by targeting B cell lymphoma 2 (BCL2) in leukemia [15]. Since then, a large number of studies have reported altered expressions of miRNA in diverse types of cancer, and the implication of those miRNAs in cancer has been investigated by loss-of-function and gain-of-function experiments in animal models and human cancer cell lines. For example, let-7 is downregulated in breast, colon, and lung cancers [16] and proven as an oncosuppressor miR to prevent tumor development by repressing RAS or MYC [17,18]. miR-34a that belongs to a p53-responsive miR-34 family was also observed to be reduced in several types of cancers. The expression level of miRNA-331-3p has been reduced in patients of nasopharyngeal carcinoma, and its overexpression induces apoptosis, resulting in the suppression of cell proliferation [19]. In contrast to those tumor-suppressive miRNAs, other miRNAs are known to be upregulated and have oncogenic roles. These include miR-21 in diverse solid tumors and hematological malignancies [20,21,22], miR-155 [23,24,25] and the miR-17~19b cluster [26,27] in B-cell lymphoma and breast cancer, and miR-106b-5p in lung cancer [28] and metastatic breast carcinoma [29].

Since miRNA expression and function are regulated upon the cellular stress [30], the limited oxygen supply, hypoxia, in the TME can affect the production and function of mature miRNAs. Epidermal growth factor receptor (EGFR) signaling is activated by the hypoxic condition to promote growth and oncogenesis [31]. Interestingly, protein argonaute 2 (AGO2) has been identified to interact with EGFR in serum-starved conditions, and deregulated AGO2 correlates with poor survival in breast cancer patients [32].

Since miRNAs are required to maintain the proper regulation of cellular processes, such as cell proliferation, cell metabolism, and protein synthesis, in normal physiological conditions, their deregulation leads to the abnormal growth and biosynthesis of cells that contribute to tumor development, progression, and metastasis (Table 1). Emerging evidence showing not only the genetic and epigenetic dysregulations of miRNA biogenesis machineries but also the regulatory mechanisms of miRNAs has demonstrated the importance of regulatory mechanisms of miRNA expression in cancer.

## 3. Dysregulation of miRNA Transcription in Cancer

Alterations of miRNA expression in cancer can arise from genomic variations of miRNA genomic loci. For example, the genomic locus of the miR-15/miR-16 cluster is deleted at high frequency in B-cell chronic lymphocytic leukemia (CLL) [72,73]. miR-146a is also repressed as a consequence of the deletion of chromosome 5q in myelodysplastic syndrome (MDS) and acute myeloid leukemia (AML) [74]. Besides the genomic variation, miRNA expression is also controlled at transcriptional level, which is mediated by transcription factors and the epigenetic control of DNA methylation.

### 3.1. Modulation of miRNA Expression by Transcription Factors in Cancer

Several studies have provided compelling evidence that alterations in transcriptional activators or repressors cause abnormal pri-miRNA transcription in cancer. For instance, expression of the *miR-34* family genes (*miR-34a*, *miR-34b,* and *miR-34c*) are controlled by the transcription factor p53 [26], reflecting the importance of the p53 functional status in predicting miR-34 expression in human cancers. Upon DNA damage and oncogenic stress, p53 is activated and regulates *miR-34* transcription, which impacts cell cycle arrest, apoptosis, and senescence [75]. miR-145 is also transcriptionally activated by upregulated p53 to induce apoptosis [33,34,76]. In contrast, the miR-143/145 cluster is repressed by oncogenic RAS signaling that induces tumorigenesis. RAS-responsive element-binding protein 1 (RREB1) leads to the transcriptional repression of the *miR-143/145* cluster, and in turn, miR-143/145 suppresses expression of RREB1, forming a tumor-promoting feedback circuit of RAS signaling [35]. In addition to p53 and RREB1, miR-145 is regulated by other transcription factors, including CCAAT/enhancer-binding protein beta (C/EBPβ), beta-catenin/T cell factor 4 (TCF4), and forkhead transcription factors FOXO1 and FOXO3 in human cancers [36,37]. The transcriptional co-factor meningioma 1 (MN1) gene is highly expressed, and its upregulation is inversely correlated with miR-20a and miR-181b transcripts in acute myeloid leukemia (AML) patients [38]. The c-Myc oncogenic transcription factor (MYC) transactivates expression of the miR-17~92 cluster (also known as oncomiR-1), and MYC-activated miR-17~92 promotes cancer progression by controlling expressions of E2F1, connective tissue growth factor (CTGF), thrombospondin 1 (THBS1), and phosphatase and tensin homolog (PTEN) in multiple cancers [39,40,41,42,43,77]. On the contrary, MYC suppresses the expression of genes of oncosuppressor miRs, such as miR-26, miR-29, miR-30, and let-7 family members in lymphoma [44,45,46]. The hypoxia-inducible factor-alpha (HIF1α) transcription factor induces the *miR-210* and *miR-155* transcription in hypoxia [48,49]. In addition, the zinc-finger E-box-binding homeobox (ZEB) transcription factors, ZEB1 and ZEB2, which are known as key activators to promote the epithelial-mesenchymal transition (EMT), repress transcription of the *miR-200* family gene [50]. It is also noted that miR-200c has been identified as a transcriptional target of MYC in nasopharyngeal carcinoma [47]. Activation protein 1 (AP1), Ets family transcription factor PU.1, C/EBPα, nuclear factor I (NFI), and signal transducer and activator of transcription 3 (STAT3) activate *miR-21* transcription by binding to the defined *miR-21* promoter [54,55]. Therefore, targeting or activating specific transcription factors responsible for the abundance of oncomiRs or oncosuppressor miRs may be promising and innovative approaches to cancer treatment.

Nuclear receptors (NRs) are ligand-activated transcription factors regulating gene expression by binding to the specific DNA sequences or regulatory regions of target genes. Since it has been reported that the NR superfamily contains 48 human members, including the hormone receptors: estrogen receptor (ER), progesterone receptor (PR), androgen receptor (AR), glucocorticoid receptor (GR), and mineralocorticoid receptor (MR) [78], several studies have shown that NRs, especially ER and AR, not only indirectly change miRNA abundance through diverse signaling pathways but also directly regulate the transcriptional activity of miRNAs in cancer. ER binds to the promoter region of the *miR-221/222* gene and recruits the NCoR/SMRT co-repressor complex to suppress miR-221/222 expression in breast cancer [51]. ER also inhibits transcription of the *miR-515*, leading to increased levels of oncogenic sphingosine kinase 1 (SK1) [52]. Like estrogen/ER, androgen/AR can regulate the transcriptional output from the miRNA loci. Indeed, numerous miRNAs have been identified to be directly regulated by androgen/AR during prostate cancer progression; these include oncomiRs, miR-125b, miR-21, miR-221/222, miR-27a, and miR-32 [53] and oncosuppressor miRs, miR-135a [56], and miR-141 [57]. The recruitment of AR to the promoter regions of these miRNAs has been demonstrated with chromatin immunoprecipitation (ChIP) analysis. In addition to ER and AR, other NRs can regulate miRNA expression in cancer. For example, PR can regulate the expression of several miRNAs, including miR-141 [58], miR-23 [59], miR-320 [60], and let-7 in human cancers [61]. Glucocorticoids have been shown to upregulate miR-15, miR-16, and miR-223 through activating both GR and MR in leukemia cell lines [62]. Therefore, increased understanding of the molecular basis of the modulation of miRNA expression by NRs may enable new therapeutic interventions for cancer patients.

### 3.2. Aberrant miRNA Expression by DNA Methylation Modification in Cancer 

In recent years, evidence has been mounting to suggest the epigenetic interaction between DNA methylation modification and miRNA expression in cancer. The transcription of pri-miRNA is also affected by epigenetic control, particularly the methylation of the promoter-associated CpG island. In human bladder cancer, *miR-127* is silenced through its promoter hypermethylation, resulting in increased expression of its cognate target, BCL6 [63]. The hypermethylation of the *miR-124-1* promoter region is also appreciated in leukemia, lymphoma, breast, colon, and liver cancers, and the epigenetic repression of the *miR-124-1* loci leads to the activation of its target, CDK6 [64,65]. The methylation of the *miR-129-2* promoter region is found in endometrial and gastric cancers, along with the upregulation of its targets, SOX4 [66]. The frequent inactivation of *miR-200* by its CpG methylayion is also found in bladder [69], breast [70], and non-small lung cancers [71]. These results suggest that DNA demethylation can activate expression of miRNAs which may act as tumor suppressors. Recently, we found that the DNA demethylase TET (ten eleven translocation) family members (TET1, TET2, and TET3) can unmask the epigenetically silenced *miR-200*, while miR-22 antagonizes miR-200 through directly targeting TETs and thereby promotes the metastatic process and EMT in breast cancer [79]. *miR-34a* and *miR-34b/c* loci are separately located on the different chromosomes among miR-34 family, but both *miR-34a* and *miR-34b/c* are hypermethylated in solid cancers and hematological tumors [67,68]. In CLL, the silencing of miRNAs by global methylation has been extensively studied using genome-wide methylation array and targeted methylation assay [80,81]. In addition to DNA methylation, histone modification has an effect on controlling miRNA expression by chromatin remodeling as well as cooperating DNA methylation modification [82]. Therefore, it is necessary to better understand how different epigenetic components interact with and influence miRNA expression and its output in the pathogenesis of cancer. 

## 4. Dysregulation of Pri-miRNA Processing in Cancer

**Figure 1:**
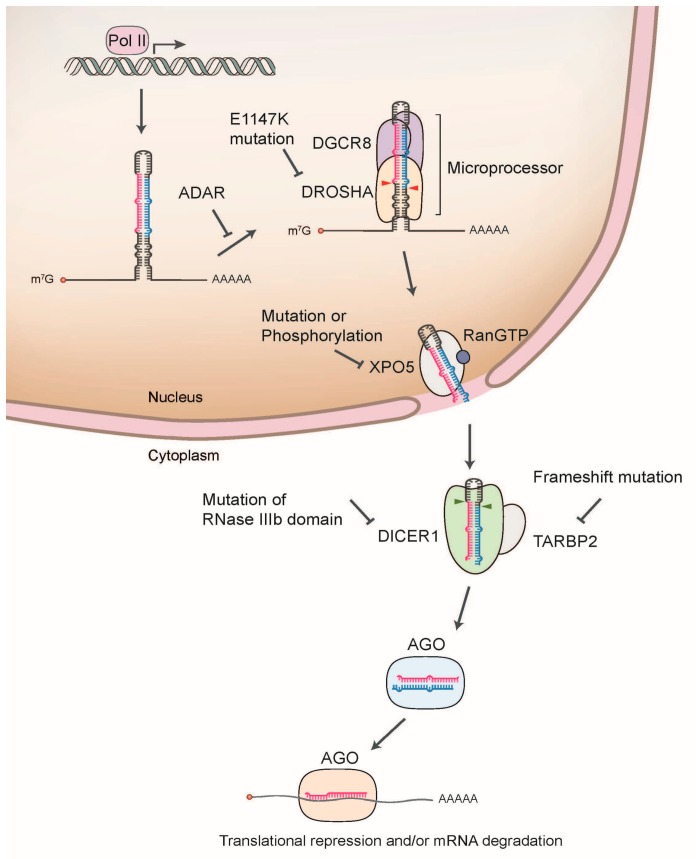
Schematic illustration of miRNA biogenesis dysregulation in cancer. The nascent primary miRNA (pri-miRNA) is transcribed by RNA Polymerase II (Pol II). The pri-miRNA is initially cleaved by DROSHA/DGCR8 microprocessor complex in the nucleus, which releases a hairpin shaped precursor (pre-miRNA). Pre-miRNA is then exported from the nucleus to the cytoplasm by exportin 5 (XPO5). In the cytoplasm, DICER1 cleaves pre-miRNA to produce the miRNA duplex, which is loaded onto the Argonaute (AGO) protein. AGO preferentially retains one strand mature miRNA and further associates with other cofactors including GW182, forming the effector complex called RNA-induced silencing complex (RISC). The RISC induces the translational suppression and mRNA degradation through the interaction with the complementary sequences in the 3’-untranslated region (3’-UTR) of target mRNA.

A class 2 ribonuclease lll enzyme, DROSHA, and its cofactor DGCR8 form a heterotrimeric complex named the “microprocessor”, which processes a stem-loop secondary structure of the nascent pri-miRNA transcript flanked by single-stranded RNA segments. The microprocessor recognizes the terminal loop region and the basal junction between the stem and the basal ssRNA segment and cleaves dsRNA at ~ 11 bp from the basal junction, releasing the hairpin-shaped pre-miRNA [83,84,85]. The aberrant processing of pri-miRNA can affect the overall production of pre-miRNA, as well as the accumulation of miscleaved pri-miRNA **(**Figure 1). In addition to the genomic mutation of the miRNA sequence, dysregulation of the microprocessor or microprocessor-associated proteins involved in pri-miRNA processing contributes to the global alterations of miRNA expression in cancer (Table 2).

### 4.1. Dysregulation of the Microprocessor in Cancer

The expression and function of the miRNA biogenesis machinery genes are often deregulated in cancer. The gain of *DROSHA* copy-number is found in more than 50% of advanced cervical squamous cell carcinomas [86], and its expression is upregulated in various types of cancers, which affect the global miRNA profile [87]. By contrast, DROSHA expression has been also shown to be downregulated in many other types of cancers, suggesting its role as a tumor suppressor in different contexts [112]. Although the function of DROSHA is still controversial, either upregulation or downregulation of *DROSHA* expression alters the global miRNA expression profile, which is correlated with cancer progression and patient survival rate [87]. *DROSHA* is frequently mutated in Wilms tumors, and mature miRNAs are globally downregulated in those tumors. The recurrent DROSHA E1147K mutation has been verified to hinder the metal binding and affect the processing activity of *DROSHA* [88,89,90,91]. It has not been yet identified in the functions of other mutations, such as the missense mutation and nonsense mutation of the *DROSHA* gene found in Wilms tumors. The expression level of DGCR8, another component of the microprocessor, has been found to be increased in various human cancers, including oesophageal, bladder, prostate, and ovarian cancers [92]. Altered expression of DGCR8 is associated with dysregulated miRNA expression and poor patient prognosis [92]. *DGCR8* is frequently mutated in Wilms tumors, and the recurrent mutation of E518K in the dsRBD1 domain of DGCR8 results in the decrease of miRNAs [89,91], implicating the importance in controlling the pri-miRNA processing machinery in cancer pathogenesis.

### 4.2. Dysregulation of the Microprocessor-Associated Proteins in Cancer

The regulators of the microprocessor, such as DROSHA- or DGCR8–associated proteins, pri-miRNA–associated RNA-binding proteins, and cellular signaling components, can also affect pri-miRNA processing. Analysis of a DROSHA-containing large complex has revealed that several microprocessor-associated RNA-binding proteins, including DEAD-box helicases p68 (also known as DDX5) [113] and p72 (also known as DDX17) [114] facilitate pri-miRNA processing. p68/p72 may serve as scaffold proteins to recruit multiple different protein factors to the DROSHA microprocessor. Interestingly, the p53 tumor suppressor protein interacts with the microprocessor complex via p68/p72 and thereby enhances the biogenesis of oncosuppressor miRs, such as miR-16-1, miR-143, and miR-145 [33]. Hippo downstream effector Yes-associated protein (YAP) also regulates pri-miRNA processing. While nuclear YAP sequesters p72, leading to p72 dissociation from the microprocessor complex to suppress miRNAs, YAP retained in the cytoplasm enable p72 to interact with the microprocessor for efficient pri-miRNA processing [115]. Likewise, constitutive activation of YAP1 or inactivation of Hippo-signaling can mediate the global downregulation of miRNAs and promote tumorigenesis. Nuclear factor 90/45 (NF90/NF45) complex impairs the access of the microprocessor to a subset of human pri-miRNAs, including pri-let-7 and pri-miR7-1, leading to the reduction in mature miRNA levels in liver cancer [93,94]. These results expand our knowledge of how pri-miRNA processing is controlled by the microprocessor regulators and cell signaling and of how this causes large perturbations of miRNA expression in cancer.

### 4.3. Dysregulation of pri-miRNA Editing in Cancer

RNA editing is a main post-transcriptional mechanism that modifies specific nucleotides at the RNA level. Adenosine deaminases acting on RNA (ADARs) are the RNA modification enzymes that convert adenosine (A) to inosine (I) in double-stranded RNAs (dsRNAs). ADAR can edit the dsRNA in the stem region of the pri-miRNA and change the secondary structure, which inhibits its processing by the DROSHA/DGCR8 microprocessor complex and leads to their degradation by endonuclease V [116]. Recent studies have demonstrated that miRNA editing is dysregulated in human cancers, and miRNA-related editing promotes or inhibits tumor development and progression [117]. Likewise, the miRNA editing level varies between different patients and cancer types (either hyperedited or hypoedited pri-miRNAs) [118,119]. The ADARs’ tissue specificity and over/underexpression in different tumor contexts may account for the diverse patterns of pri-miRNA editing in cancer. Nevertheless, the pathophysiological role of pri-miRNA editing events observed in cancer remains largely unexamined.

## 5. Dysregulation of Pre-miRNA Processing in Cancer

Pre-miRNA generated by the microprocessor in the nucleus is transported into the cytoplasm by a complex of XPO5 and RAN-GTP, a cofactor of XPO5. It is further processed to generate ~ 22 nt small RNA duplexes. DICER1 recognizes 2 nt 3′ overhang of pre-miRNA, 22 nt apart from which the cleavage site is defined [120]. DICER1 associates with the dsRNA-binding protein TARBP2 to increase the stability of the DICER1-RNA complex and enhance the fidelity of miRNA processing. Importantly, genetic mutations and dysregulation of key components in the pre-miRNA processing step cause aberrant miRNA expression in cancer.

### 5.1. Defect in Pre-miRNA Export in Cancer

Inactivated mutations of XPO5 have been identified in sporadic colon, gastric, and endometrial tumors with microsatellite instability [95]; these mutations cause the defect of pre-miRNA export, leading to the accumulation of pre-miRNA in the nucleus. The genetic alterations of XPO5 are also associated with the risk of breast cancer [121]. Additionally, the MAPK/ERK pathway can suppress pre-miRNA export through phosphorylating XPO5 at Thr345, Ser416, and Ser497 [96]. Phosphorylation of XPO5 correlates with the global downregulation of miRNAs and poor prognosis in patients with hepatocellular carcinoma, providing functional and clinical evidence of the cancer-associated dysregulation of XPO5 for aberrant miRNA processing and tumorigenesis. However, the upstream signaling regulators for pre-miRNA export, via either XPO5 or Ran-GTP, have not yet been identified.

### 5.2. Dysregulation of DICER1 and TARBP2 in Cancer

Global inhibition of miRNA biogenesis by depletion of DICER1 promotes cell growth and tumorigenesis in human cancer cell lines and mouse models of cancer [122], suggesting the oncogenic role of DICER1 in tumorigenesis. Recurrent somatic and germline DICER1 mutations that change its protein levels and/or impair its function, leading to defective pre-miRNA processing, are frequently found in many types of tumors, including pleuropulmonary blastoma, rhabdomyosarcoma, non-epithelial ovarian cancer, and liver tumor [97,98,99,100,101,102]. In particular, mutations within the RNase IIIb domain of DICER1 markedly reduce the expression of 5p miRNAs (miRNAs derived from the 5′ side of the pre-miRNA) in cancer [92,103]. DICER1-associated regulatory factors are also involved in the dysregulation of pre-miRNA processing. TAp63 suppresses tumorigenesis and metastasis by direct binding to DICER [123], suggesting both genetic mutation and functional inactivation of DICER1 dictate global miRNA expression in tumor malignancy.

The frameshift mutations of TARBP2 are found in sporadic and hereditary carcinomas with microsatellite instability, which correlates with reduced levels of DICER1 and mature miRNAs [106,107]. TARBP2 is also deleted in 15% of adenoid cystic carcinoma [104]. In contrast, TARBP2 is overexpressed in cutaneous melanoma, adrenocortical carcinoma, and metastatic breast and prostate cancers [105], suggesting its specific pivotal role in different cancer types.

### 5.3. Dysregulation of AGO2 in Cancer

Argonaute 2 (AGO2), the only member of the Argonautes with an intrinsic endonuclease activity, is involved in the accumulation of mature miRNAs [124,125]. As a key regulator of miRNA function and maturation, AGO2 has been found to be overexpressed in various types of human cancers, including breast, gastric, and head and neck cancers [108,109,110,111]. The overexpression of AGO2 may facilitate oncomiRs to repress their targets [126]. Despite identified functions of AGO2 in different types of cancer being contradicted [127], its dysregulation has been implicated in recent years in tumorigenesis.

## 6. Conclusions

Numerous studies have documented the aberrant expression of miRNAs in cancer and the oncogenic or tumor-suppressor roles of miRNAs. Likewise, the regulatory mechanisms to control the expression of miRNAs are strongly associated with cancer diagnosis, prognosis, and treatment, as well as the pathogenesis of cancer. Different core players and their partners involved in the multiple sequential step process for producing miRNA show deregulated activity and abundance in cancers, some of which are known to be affected by cancer-associated signaling regulators. Nevertheless, current knowledge is still behind a comprehensive understanding of how each miRNA is specifically controlled in specific types of cancer, emphasizing the systemic approach to the multi-layered regulation governing miRNA expression in cancers. We have discussed the series of processes to generate miRNAs and the possible regulatory mechanisms modulating miRNA expression in cancers. Dysregulation of miRNA biogenesis inevitably changes the mRNA profile in a cell, which in turn affects the miRNA expression and function through a feedback loop. Thus, it is necessary to comprehensively investigate the gene expression regulatory networks that cover both miRNA expression and its effect on mRNA targets in the near future. With evolved technologies such as the gene editing system by CRISPR-Cas9 and high-throughput sequencing, the studies to understand the molecular and cellular regulatory mechanisms controlling the expression of miRNAs in cancer will be facilitated and suggest compelling evidence to explore new therapeutic strategies for the treatment of cancer by targeting or restoring the expression profiles of miRNAs.

## Figures and Tables

**Table 1:** Dysregulation of miRNA transcription in cancer.

**Regulation of miRNA Expression by DNA Binding Factor**				
**Factor**	**miRNA**	**Mechanism/Function/** **Clinical Correlation**	**Cancer Type**	**References**
Transcriptional activation by p53	miR-34a, miR-34b miR-34c	Cell cycle arrest, apoptosis & senescence	Various types of cancers	[26]
	miR-145	Apoptosis	Various cancers like prostate cancer	[33,34]
Transcriptional repression by RREB1	miR-143/145 cluster	Transcriptional repression of miR-143/145 cluster	Various cancers like Pancreatic, Colorectal Adenocarcinoma	[35]
Regulation by C/EBPβ, beta-catenin/TCF4, FOXO1 & FOXO3	miR-145		Various cancers like Renal cancer	[36,37]
Regulation by MN1	miR-20a, miR-181b	Inverse correlation between MN1 and miRNAs	acute myeloid leukemia (AML) patients	[38]
Transcriptional activation by Myc	miR-17~92 cluster	Controls the expression of E2F1, THBS1, CTGF, & PTEN	Various types of cancer, including B- Cell lymphoma & Breast cancer	[39,40,41,42,43]
	miR-200c, miR-26, miR-29, miR-30, let-7	Suppresses the expression of their genes	Nasopharyngeal carcinoma & Lymphoma	[44,45,46,47]
HIF1α	miR-210	Repression of initiation of tumor growth	Various cancers like Head & neck tumor	[48]
	miR-155			[49]
ZEB1 & ZEB2	miR-200 family		Various cancers	[50]
Repression by ER	miR-221/222	Suppression of miR-221/222 expression by NcoR/SMRT complex	Breast cancer	[51]
	miR-515	Increased levels of oncogenic SK1	Breast cancer	[52]
Androgen/AR	miR-125b, miR-21, miR-221/222, miR-27a, miR-32	Oncogenic role	Prostate cancer & Hematological malignancies	[53] [54,55]
	miR-135a, miR-141	Tumor suppressive role	Prostate cancer	[56,57]
Progesterone receptor /PR	miR-141, miR-23, miR-320, let-7		Breast & ovarian cancer	[58,59,60,61]
Glucocorticoids/ GR	miR-15, miR-16, miR-223	Incresead expression of miRNA	Leukemia cell lines	[62]
**Regulation of miRNA Expression by Epigenetic Alteration**				
**Factor**	**miRNA**	**Mechanism/Function/** **Clinical Correlation**	**Cancer Type**	**References**
Promoter hypermethylation	miR-127	Increassed expression of BCL6	Bladder Cancer	[63]
Promoter hypermethylation	miR-124-1	Activation of , CDK6	Breast, Colon, Liver, Leukemias & Lymphomas	[64,65]
Promoter hypermethylation	miR-129-2	Upregulation of SOX4	Endometrial Gastric cancer	[66]
Promoter hypermethylation	miR-34a miR-34b/c		Gastric, Prostate & Colon cancer	[67,68]
CpG methylation	miR-200	Inactivation	Bladder, breast, non-small lung cancer, leukemia	[69,70,71]

**Table 2:** Dysregulation of miRNA biogenesis.

**The Microprocessor in Cancer**				
**Factor**	**miRNA**	**Mechanism/Function/** **Clinical Correlation**	**Cancer Type**	**References**
Up/downregulation of DROSHA	Global miRNA expression	Cancer progression & poor patient survival	Cervical carcinoma, Wilms tumor	[86,87]
Drosha E147K mutation	Global miRNA expression	Reduced function	Wilms tumors	[88,89,90,91]
Upregulation of DGCR8 expression	Global miRNA expression	Dysregulation is associated with poor patient survival	Esophageal, Bladder, Prostate & ovarian cancer	[92]
E518K mutation in the dsRBD1 domain of DGCR8	Decrease of crucial miRNAs		Wilms tumors	[89,91]
**Regulation of Microprocessor in Cancer**				
**Factor**	**miRNA**	**Mechanism/Function/** **Clinical Correlation**	**Cancer Type**	**References**
NF90/NF45	pri-let-7, pri-miR-7-1	Inhibits the processing	Hepatocellular carcinoma	[93,94]
**Pre-miRNA Export in Cancer**				
**Factor**	**miRNA**	**Mechanism/Function/** **Clinical Correlation**	**Cancer Type**	**References**
Mutations of XPO5	Global miRNA expression	Accumulation of pre-miRNA in the nucleus	Sporadic colon cancer, Gastric & Endometrial cancer	[95]
Phosphorylation of XPO5 at Thr345, Ser416, and Ser497	Global miRNA expression	Correlates with global miRNA downregulation and with poor survival in patients	Hepatocellular carcinoma,	[96]
**DICER1 and TARBP2 in Cancer**				
**Factor**	**miRNA**	**Mechanism/Function/** **Clinical Correlation**	**Cancer Type**	**References**
Mutations of DICER1	Global miRNA expression	Somatic and germline DICER1 mutations lead to defective pre-miRNA processing	Pleuropulmonary blastoma, Rhabdomyosarcoma, non-epithelial ovarian cancers, liver tumor	[97,98,99,100,101,102]
Mutations within the RNase IIIb domain of DICER1	5p miRNAs	Deregulation of pre miRNA expression	Various cancer like ovarian cancer	[92,103]
TARBP2 Deletion	Global miRNA expression		Adenoid cystic carcinoma	[104]
Upregulation of TARBP2 expression			Melanoma, breast & prostate cancer	[105]
Frameshift mutations of TARBP2	Global miRNA expression	Reduced levels of DICER1 and mature miRNAs	Sporadic & hereditary carcinomas	[106,107]
**AGO2 in Cancer**				
**Factor**	**miRNA**	**Mechanism/Function/** **Clinical Correlation**	**Cancer Type**	**References**
AGO2 expression dysregulation	oncomiRs	Repression of the targets of oncomiRs	Breast, gastric, head & neck cancers	[108,109,110,111]

## Abbreviations

mRNA	Messenger RNA
miRNA	MicroRNA
pri-miRNA	Primary-microRNA
pre-miRNA	Precursor-microRNA
XPO5	Exportin 5
AGO2	Argonaute 2
TNRC6A	Trinucleotide Repeat Containing Adaptor 6A
RISC	RNA-induced Silencing Complex
miRISC	miRNA-induced silencing complex
3′-UTR	3′- Untranslated Region
DNA	Deoxyribonucleic Acid
CLL	Chronic Lymphocytic Leukemia
BCL2	B-cell Lymphoma 2
MDS	Myelodysplastic syndrome
AML	Acute Myeloid Leukemia
RREB1	Ras Responsive Element Binding Protein 1
C/EBPβ	Ras Responsive Element Binding Protein 1
C/EBPβ	CCAAT-enhancer-binding Protein Beta
TCF4	Transcription Factor 4
FOXO1	Forkhead Box Protein O1
FOXO3	Forkhead Box Protein O3
MN1	Meniongoma-1
E2F1	E2F Transcription Factor 1
CTGF	Connective Tissue Growth Factor
THBS1	Thrombospondin 1
PTEN	Phosphatase and Tensin Homolog
HIF1-α	Hypoxia-inducible Factor 1-alpha
ZEB	Zinc Finger E-box-binding Homeobox 1
EMT	Epithelial-Mesenchymal Transition
NRs	Nuclear Receptors
ER	Estrogen Receptor
GR	Glucocorticoid Receptor
MR	Mineralocorticoid Receptor
NCoR/SMRT	Nuclear Receptor co-repressor/ Silencing Mediator of Retinoic
SK1	Sphingosine Kinase 1
ChIP	Chromatin Immunoprecipitation
BCL6	B-cell Lymphoma 6
CDK6	Cyclin-dependent Kinase 6
SOX4	SRY-Box Transcription Factor 4
CDH1	Cadherin-1
TET	Ten-eleven Translocation Methylcytosine Dioxygenase
PLAG	Pleomorphic adenoma gene
CREB	cAMP-response element binding
DGCR8	DiGeorge Critical Region 8
ssRNA	Single-stranded RNA
dsRNA	Double-stranded RNA
dsRBD1	dsRNA Binding Domain1
DDX5	DEAD-box Helicases p68
DDX17	DEAD-box Helicases p72
YAP	Yes-associated Protein
NF90/NF45	Nuclear Factor 90/45
ADAR	Adenosine Deaminases Acting on RNA
RAN-GTP	RAs-related Nuclear protein-GTP
TARBP2	Tar RNA-Binding Protein 2
CRISPR	Clustered Regularly Interspaced Short Palindromic Repeats
